# Recognition of the Script in Serbian Documents Using Frequency Occurrence and Co-Occurrence Analysis

**DOI:** 10.1155/2013/896328

**Published:** 2013-12-10

**Authors:** Darko Brodić, Zoran N. Milivojević, Čedomir A. Maluckov

**Affiliations:** ^1^Technical Faculty in Bor, University of Belgrade, Vojske Jugoslavije 12, 19210 Bor, Serbia; ^2^Technical College Niš, Aleksandra Medvedeva 20, 18000 Niš, Serbia

## Abstract

Any document in Serbian language can be written in two different scripts: Latin or Cyrillic. Although characteristics of these scripts are similar, some of their statistical measures are quite different. The paper proposed a method for the extraction of certain script from document according to the occurrence and co-occurrence of the script types. First, each letter is modeled with the certain script type according to characteristics concerning its position in baseline area. Then, the frequency analysis of the script types occurrence is performed. Due to diversity of Latin and Cyrillic script, the occurrence of modeled letters shows substantial statistics dissimilarity. Furthermore, the co-occurrence matrix is computed. The analysis of the co-occurrence matrix draws a strong margin as a criteria to distinguish and recognize the certain script. The proposed method is analyzed on the case of a database which includes different types of printed and web documents. The experiments gave encouraging results.

## 1. Introduction

Cryptography studies the problems concerning the conversion of information from a readable to some other state. It deals with information which is changing from one to another state. The initial information represents a plain text. When the information becomes encrypted, it is referred as a cipher text [1]. A substitution cipher is a method of encoding. According to it, the units of plain text are replaced with cipher text [2]. They can be single letters, pairs of letters, triplets of letters, mixtures of the above, and so forth. In our application the encryption function is not needed to be injective [3] due to nature of further statistical analysis. It does not matter if it will encrypt two different plain texts into the same cipher text, because decryption of the cipher text is not considered. Hence, the cryptography is used only as a basis for modeling and analyzing documents written in Serbian language. Serbian language represents the European minority language. However, it is distinct due to its capability to be written in Latin and Cyrillic script, interchangeably. According to the baseline characteristics [4], each letter in the text file is replaced with the cipher which is taken from the set of four counterparts only. The basic idea is to distinguish the script (Latin or Cyrillic) according to statistical analysis of the cipher text. It is accomplished with frequency analysis concerning occurrence [5] as well as with the method using statistical measures extracted from gray-level co-occurrence matrix [6]. The letter frequency distribution is a function which assigns each letter a frequency of its occurrence in the text sample [7]. The gray-level co-occurrence matrix (GLCM) have used for the extraction of features needed for texture classification [8]. Nevertheless, it can be exploited for a letter co-occurrence in a text document [9]. At the final stage, the experiment is made on a custom oriented database containing text from printed and Web documents.

The rest of the paper is organized as follows.  Section 2 describes the full procedure of the proposed algorithm.  Section 3 defines the experiment.  Section 4 presents the results from experiment and discusses them.  Section 5 makes a conclusion.

## 2. Proposed Algorithm

**Figure 1:**
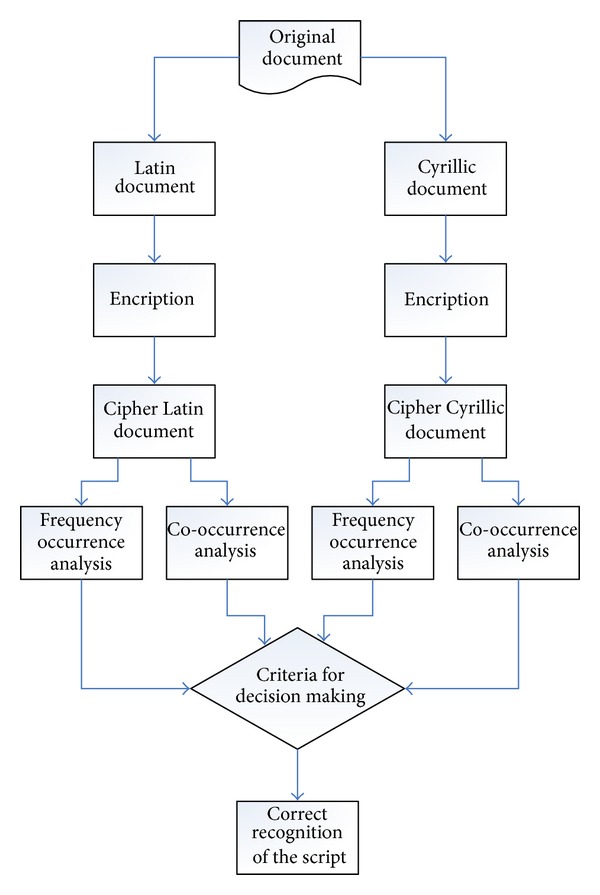
The flow of proposed algorithm.

The proposed algorithm converts document written in Latin and Cyrillic script which represent the plain text into cipher text according to predefined encryption based on text line structure definition. Then, the equivalent cipher texts are subjected to the frequency and co-occurrence analysis. The results of frequency analysis indicated a substantial difference between cipher texts obtained from Latin and Cyrillic text. Similarly, co-occurrence analysis shows obvious quantitative disparity in some measures. This draws a strong margin as a criterion in order to distinguish and recognize a certain script type (Figure 1).

### 2.1. Text Line Structure

**Figure 2:**
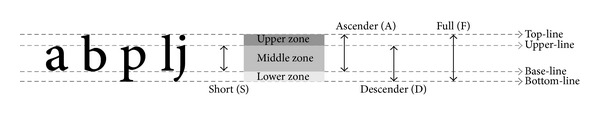
Definitions of the script characteristics.

Text in printed and Web documents is defined as well-formed text type. It is characterized by strong regularity in shape. The distances between the text lines are adequate to be split up. The words are formed regularly with similar distance. Their inter word spacing is decent as well. However, in certain script, the letters or signs have different position according to its baseline. It is shown in Figure 2.

From Figure 2 four virtual lines can be defined [4]:

(i) The top-line,
(ii) The upper-line,
(iii) The base-line, and
(iv) The bottom-line.

Accordingly, a text line can be considered as being composed of three vertical zones [4]:

(i) The upper zone,
(ii) The middle zone, and
(iii) The lower zone.

Each text line has at least a middle zone. The upper zone depends on capital letters and letters with ascenders, while the lower zone depends on letters with descenders. Only a few letters occupy the upper and lower zone.

### 2.2. Encryption

Two different sets are produced. They are *A*_*L*_ and *A*_*C*_ for Latin and Cyrillic alphabet, respectively:

(1) $$\begin{array}{c} A_{L}=\left\{\text{A},\text{B},\text{C},\ldots ,\overset{ˇ}{\text{Z}},\text{a},\text{b},\text{c},\ldots ,\overset{ˇ}{\text{z}}\right\},\\ A_{C}=\left\{\text{A},Б,Ц,\ldots ,Ш,\text{a},б,ц,\ldots ,ш\right\}. \end{array}$$

Each of them consists of 60 elements that is, letters, which are valid for Serbian language. Furthermore, both sets *A*_*L*_ and *A*_*C*_ are mapped into set *C*.

(2) $$\begin{array}{c} f_{L}:A_{L}\mapsto C,\\ f_{C}:A_{C}\mapsto C. \end{array}$$

These mappings are achieved in accordance with the text line area definition. The structure of text line allows definition of following script types [4].

(i) Full letter (F), where letter is present in all three zones.
(ii) Ascender letter (A), where character parts are present in the upper and middle zones.
(iii) Descender letter (D), where character parts are present in the lower and middle zones, and
(iv) Short letter (S), where character parts are present in the middle zone only.

Accordingly, all letters will be replaced with the cipher from the following set:

(3) $$\begin{array}{c} C=\left\{\text{S},\text{A},\text{D},\text{F}\right\}. \end{array}$$

All letters can reach certain position, which belongs to set *C* with a unique designation according to Table 1.

It should be noted that above mappings are surjective.

Serbian language contains 30 letters. Each letter in Latin has a corresponding equivalent letter in Cyrillic.  Table 2 shows Latin and Cyrillic letters as well as theirs designation according to Table 1.

Statistical analysis of the letters and theirs corresponding type for Latin and Cyrillic scripts is shown in Table 3.

### 2.3. Frequency Analysis of the Occurrence

**Figure 3:**
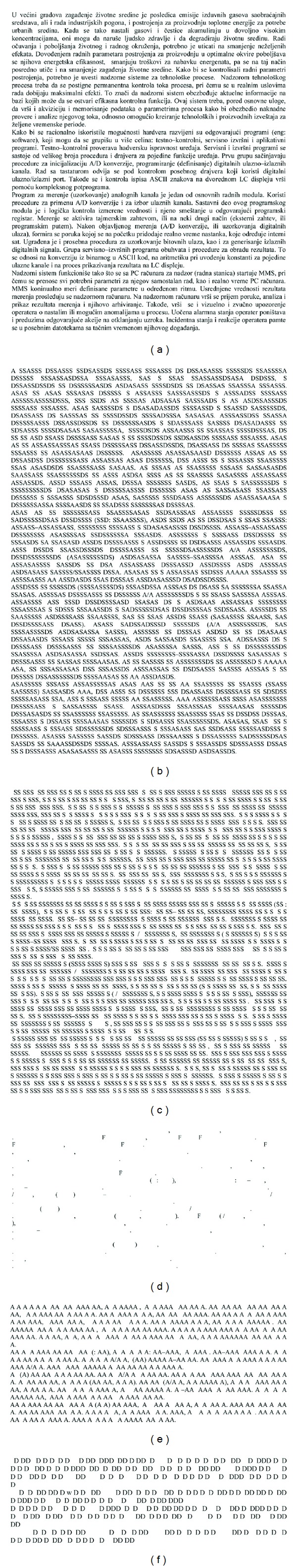
Application of the proposed algorithm: (a) Original Latin text, (b) Cipher text according to set *C*, (c) Only “S” text, (d) Only “F” text, (e) Only “A” text, (f) Only “D” text.

In the proposed algorithm, all letters from certain script has been substituted with equivalent members of the set *C* according to Table 2. These circumstances for Latin document are shown in Figure 3.

**Figure 4:**
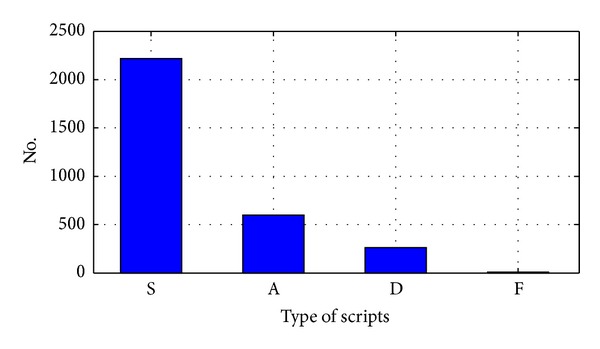
Distribution of *C* set's elements in cipher text obtained from Latin document.

Figure 3(b) shows the cipher text which is obtained from Latin documents according to modeling given in Table 2. Figures 3(c)–3(f) shows a subset of cipher text with each element of set *C*, that is, S, A, D and F, respectively. Statistical analysis of the cipher text shows following: 2217 elements of S, 598 elements of A, 261 elements of D and 8 elements of F types. Accordingly, distribution of *C* set elements for Latin document is shown in Figure 4.

**Figure 5:**
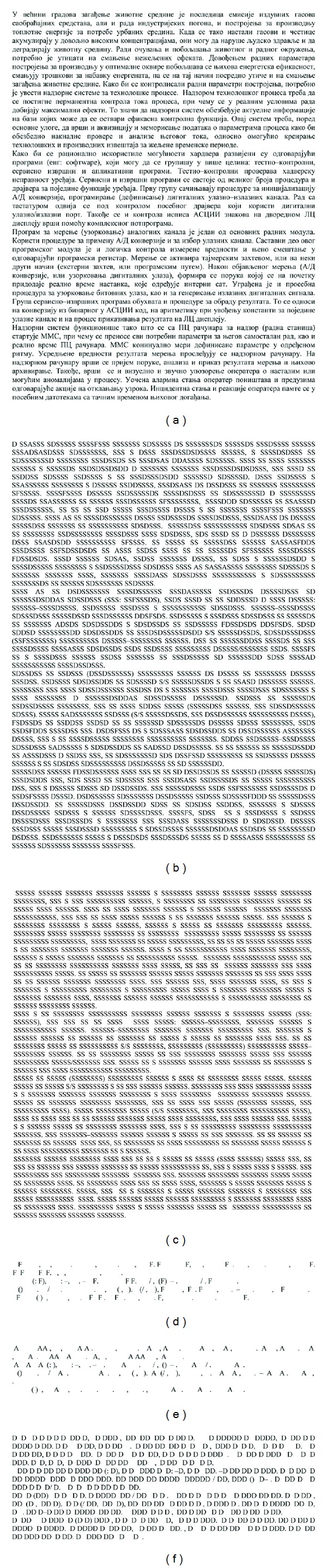
Application of the proposed algorithm: (a) Original Cyrillic text, (b) Cipher text according to set *C*, (c) Only “S” text, (d) Only “F” text, (e) Only “A” text, (f) Only “D” text.

Currently, the same Latin document is converted into Cyrillic one. Similarly as in Latin document, all letters from Cyrillic document are exchanged with the equivalent members of the set *C* according to Table 2. These circumstances for Cyrillic document are shown in Figure 5.

Figure 5(b) shows the cipher text which is obtained from Cyrillic documents according to modeling given in Table 2. Figures 5(c)–5(f) shows a subset of cipher text with each element of set *C*, that is, S, A, D and F, respectively. 

**Figure 6:**
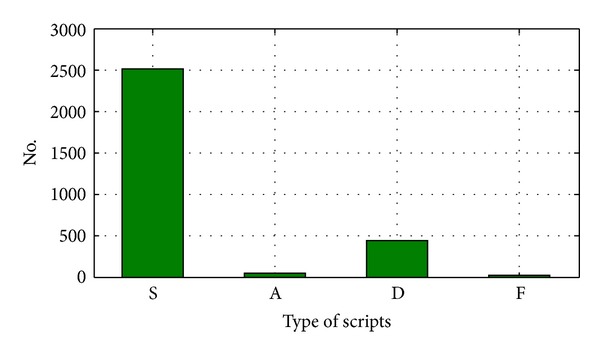
Distribution of *C* sets elements in cipher text obtained from Cyrillic document.

Statistical analysis of the Cyrillic document image shows following: 2516 elements of S, 53 elements of A, 445 elements of D and 26 elements of F types. It should be noted that the sum of all *C* set elements in Latin and Cyrillic document is not quite identical. It is valid due to difference in definition of letters in two scripts. In the Cyrillic script, each letter is given one and only one sign. However, in Latin script letters such as dž, lj and nj are represented by two letters. Distribution of *C* set elements for Cyrillic document is presented in Figure 6.

**Figure 7:**
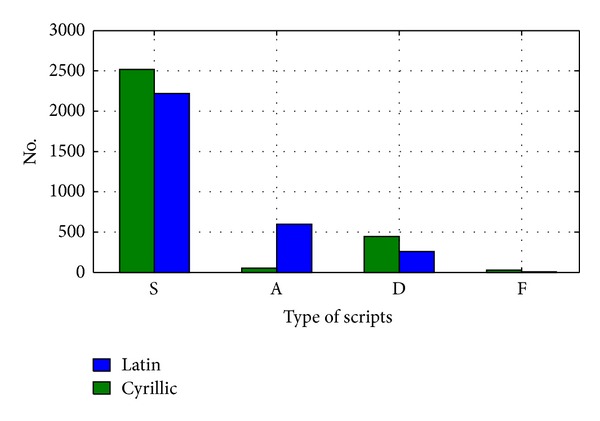
Comparison between distributions of *C* set elements in Latin and equivalent Cyrillic document.

According to Figures 4 and 6, the comparison chart is drawn. It is shown below in Figure 7.

Quantification of the script type appearance in a document written in Latin and Cyrillic is shown in Table 4.

It is obvious that the Latin document compared to Cyrillic one has slightly smaller number of short (S), descender (D) and full (F) letters. Nonetheless, the crucial margin is seen in ascender (A) letters. Hence, it can be a measure of confidence for detection of the script in a document given in Serbian language. 

### 2.4. Co-Occurrence Analysis

Let **I** be the gray scale image which is under consideration. It has *M* row and *N* columns, while *T* is the total number of gray levels. The spatial relationship of gray levels in the image **I** is expressed by the grayscale co-occurrence matrix (GLCM) **C** [6, 10]. Hence, **C** is a matrix that describes the frequency of one gray level appearing in a specified spatial linear relationship with another gray level within the area of investigation [11]. In order to compute a co-occurrence matrix **C**, we considered a central pixel *I*(*x*, *y*) with a neighborhood defined by the window of interest. This window is defined by two parameters: inter-pixel distance (*d*) and orientation (*θ*). Typically, the choice of *d* is 1 (one pixel), while the value of *θ* depends on the neighborhood. Because of that, each pixel has 8 neighbors given at following angles *θ* = 0°, 45°, 90°, 135°, 180°, 225°, 270°, 315°. However, the case of neighbors at *θ* = 0° or at *θ* = 180° is similar to the GLCM definition [12]. So, the choice may fall to 4 neighbors pixels at *θ* = 0°, 45°, 90° and 135°, that is, horizontal, right diagonal, vertical and left diagonal [13]. These orientations refer to 4-adjacent pixels at (*x* + *d*, *y*), (*x*, *y* − *d*), (*x* − *d*, *y*) and (*x*, *y* + *d*), where *d* is 1. For each pixel of the neighborhood, it is counted the number of times a pixel pair appears specified by the distance, and orientation parameters. The (*i*, *j*)th entry of **C** represents the number of occasions a pixel with an intensity *i* is adjacent to a pixel with an intensity *j*. Hence, for the given image **I**, the co-occurrence matrix **C** is defined as [14]:

(4) $$\begin{array}{c} C\left(i,j\right)=\overset{M}{\underset{x=1}{\sum }}\;\overset{N}{\underset{y=1}{\sum }}\{\begin{array}{cc} 1, & \begin{array}{c} \text{if}\;\;I\left(x,y\right)=i,\\ I\left(x+\Delta x,y+\Delta y\right)=j \end{array}\\ 0, & \text{otherwise}, \end{array} \end{array}$$

where *i* and *j* are the image intensity values of the image, *x* and *y* are the spatial positions in the image **I**. The offset (Δ*x*, Δ*y*) is specifying the distance between the pixel-of-interest and its neighbor. It depends on the direction *θ* that is used and the distance *d* at which the matrix is computed. The square matrix **C** is of the order *N*. Using a statistical approach like GLCM provides a valuable information about the relative position of the neighboring pixels in an image [12]. In order to normalize matrix **C**, matrix **P** is calculated as [10]:

(5) $$\begin{array}{c} P\left(i,j\right)=\frac{C\left(i,j\right)}{\sum_{i=1}^{N}\sum_{j=1}^{N}C\left(i,j\right)}. \end{array}$$

The normalized co-occurrence matrix **P** is obtained by dividing each element of **C** by the total number of co-occurrence pairs in **C**. 

**Figure 8:**
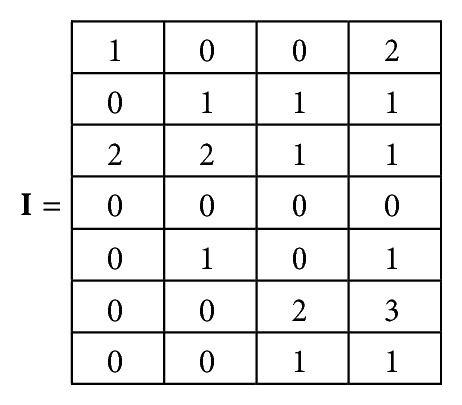
Initial 4-level grayscale matrix **I** (featuring *M* = 7, *N* = 4, and *L* = 4).

To illustrate the computing of GLCM, a four gray level image **I** is used. The window parameters are *d* = 1 and *θ* = 0° (horizontal). Initial matrix **I** is shown in Figure 8.

**Figure 9:**
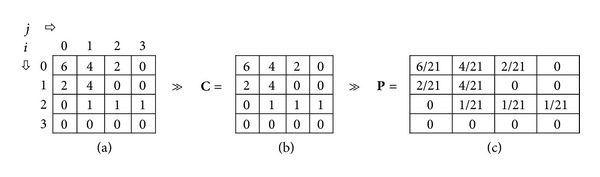
Co-occurrence matrix for grayscale matrix **I** (*d* = 1 and *θ* = 0°): (a) The number of occasions a pixel with an intensity *I* is adjacent to a pixel with intensity *j*, (b) Co-occurrence matrix **C**, (c) Normalized matrix **P**.

The procedure of calculating co-occurrence matrix for grayscale matrix **I** (*d* = 1 and *θ* = 0°) [12] is given in Figure 9. 

**Figure 10:**
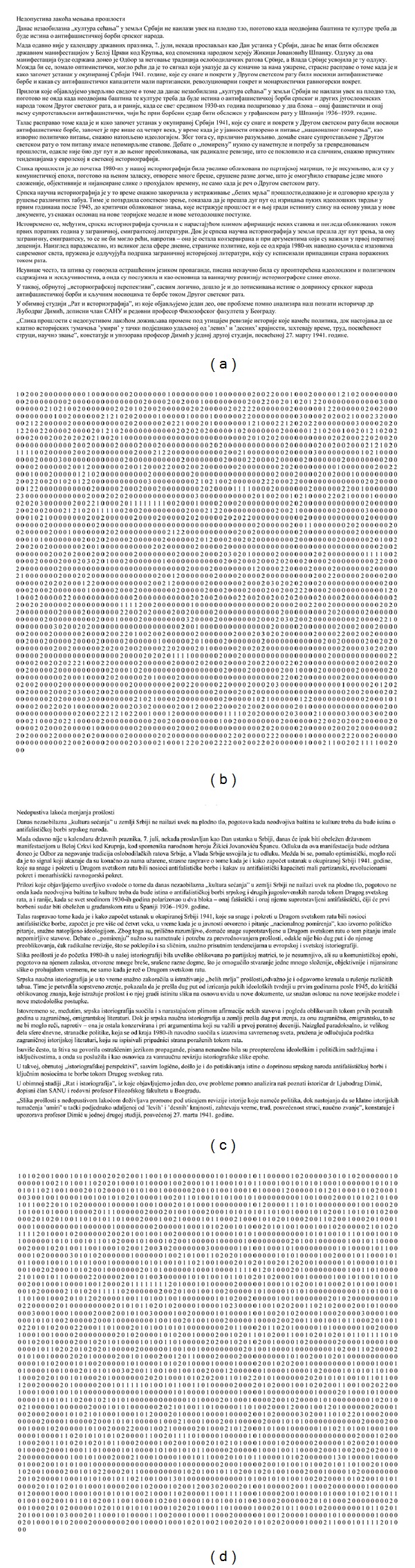
Document conversion: (a) Original Cyrillic text, (b) Cipher text obtained from Cyrillic text according to set *C*_*N*_, (c) Original Latin text (equivalent to Cyrillic one), (d) Cipher text obtained from Latin text according to set *C*_*N*_.

In order to GLCM be applied in our case, set *C* is mapped into set *C*_*N*_ by bijective function as:

(6) $$\begin{array}{c} f_{C}:C\mapsto C_{N}, \end{array}$$

where *C*_*N*_ = {0,1, 2,3}. Furthermore, the neighborhood is given as 2-connected (*x* − *d* and *x* + *d* around *x*, where *d* = 1). According to that, the same document in Latin and Cyrillic script is converted into cipher text. It is shown below in Figure 10.

To evaluate these cipher documents GLCM method is employed. Nevertheless, various statistic measures obtained from the co-occurrence matrix is introduced. The primary goal is to characterize the cipher text. Five descriptors can be used to describe the image [15]:

(i) Uniformity (UNI),
(ii) Entropy (ENT),
(iii) Maximum probability (MAX),
(iv) Dissimilarity (DIS), and
(v) Contrast (CON).

Uniformity (UNI) which is sometimes called angular second moment (ASM) or energy (ENG) measures the image homogeneity. It receives the highest value when GLCM has few entries of large magnitude. In contrast, it is low when all entries are nearly equal. The equation of the uniformity is [15]:

(7) $$\begin{array}{c} \text{UNI}=\overset{N}{\underset{i=1}{\sum }}\overset{N}{\underset{j=1}{\sum }}P{\left(i,j\right)}^{2}. \end{array}$$

Entropy (ENT) measures the disorder or the complexity of the image. The highest value is found when the values of *P*(*i*, *j*) are allocated quite uniformly throughout the matrix. This happens when the image has no pairs of gray level, with particular preference over others. The equation of the entropy is [15, 16]:

(8) $$\begin{array}{c} \text{ENT}=-\overset{N}{\underset{i=1}{\sum }}\overset{N}{\underset{j=1}{\sum }}P\left(i,j\right)\cdot \log P\left(i,j\right). \end{array}$$

Maximum probability (MAX) extracts the most probable difference between gray scale value in pixels. It is defined as [15]:

(9) $$\begin{array}{c} \text{MAX}=\max\left\{P\left(i,j\right)\right\}. \end{array}$$

Dissimilarity (DIS) is a measure of the variation in gray level pairs of the image. It depends on distance from the diagonal weighted by its probability. The equation of the dissimilarity is [15]:

(10) $$\begin{array}{c} \text{DIS}=\overset{N}{\underset{i=1}{\sum }}\overset{N}{\underset{j=1}{\sum }}P\left(i,j\right)\cdot \left|i-j\right|. \end{array}$$

Contrast (CON) or inertia is a measure of the intensity contrast between a pixel and its neighbor over the entire image. Hence, it shows the amount of local variations present in the image. If the image is constant, then the contrast will be equal 0. The highest value of contrast is obtained when the image has random intensity and the pixel intensity and neighbor intensity are very different. The equation of the contrast is [15, 16]:

(11) $$\begin{array}{c} \text{CON}=\overset{N}{\underset{i=1}{\sum }}\overset{N}{\underset{j=1}{\sum }}P\left(i,j\right)\cdot {\left(i-j\right)}^{2}. \end{array}$$

A brief look at the normalized co-occurrence matrix **P** for the same document written in Latin and Cyrillic scripts (text representing the excerpt of the first four paragraphs from a document given in Figure 10) shows quite a different characterization. The test results are given in Table 5.

Furthermore, the calculation of five co-occurrence descriptors shows the values given in Table 6.

## 3. Experiments

**Figure 11:**
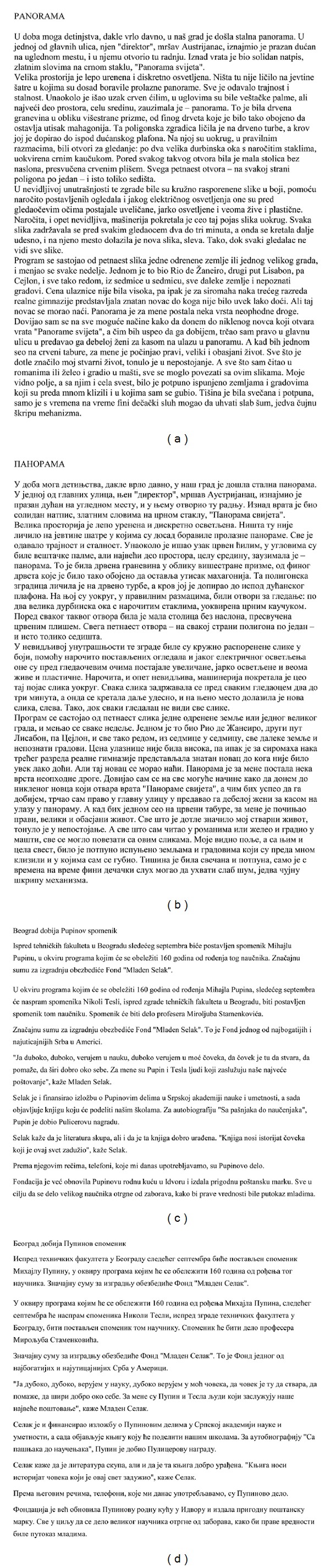
Custom-oriented database: (a) Printed document in Latin, (b) Printed document in Cyrillic, (c) Web document in Latin, (d) Web document in Cyrillic.

For the sake of the experiment, a custom-oriented database is created. It consists of 10 documents. These documents represent excerpts from printed and web documents written in Serbian language. The documents are created in both scripts: Latin and Cyrillic. Printed documents are created from PDF documents, while web documents are extracted from web news. The total length of documents given in the database is approx. 75000 letter characters per script (approx. 40 pages). The length of printed documents is from 2273 to 15840 letter characters. Web documents are smaller compared to printed documents. Their length is from 1231 to 2502 letter characters. It should be noted that all documents have more than 1000 letter characters. The example of the printed and web document from the database is shown in Figure 11.

## 4. Results and Discussion

According to the proposed algorithm, all documents from the database are converted into equivalent cipher texts and subjected to the frequency and co-occurrence analysis. First, the frequency analysis of the script type occurrence in Latin as well as in Cyrillic documents is examined (Table 7). The obtained results for each document are given in Table 8.

The final processing of the results is based on cumulative measures like sum, average, max and min of script type occurrence in the database. According to that the criteria are established. All these are shown in Table 9. 

From cumulative results given in Table 10 some criteria can be established. It can be noted that the biggest margin between results are seen in the ratio of ascending letters. This ratio has the value of at least 8. Hence, it is the strongest point of qualitative characterization and recognition of the certain script. Furthermore, the smaller number of short and descending scripts are common in Latin compared to Cyrillic documents. At the and, full letters are quite rare in a Latin document. However, its characterization in criteria form is quite problematic due to their absence in Latin documents from time to time.

Furthermore, the analysis of the script type co-occurrence in Latin as well as in Cyrillic documents is examined according to GLCM method. The obtained results for each document are given in Table 10.

**Figure 13:**
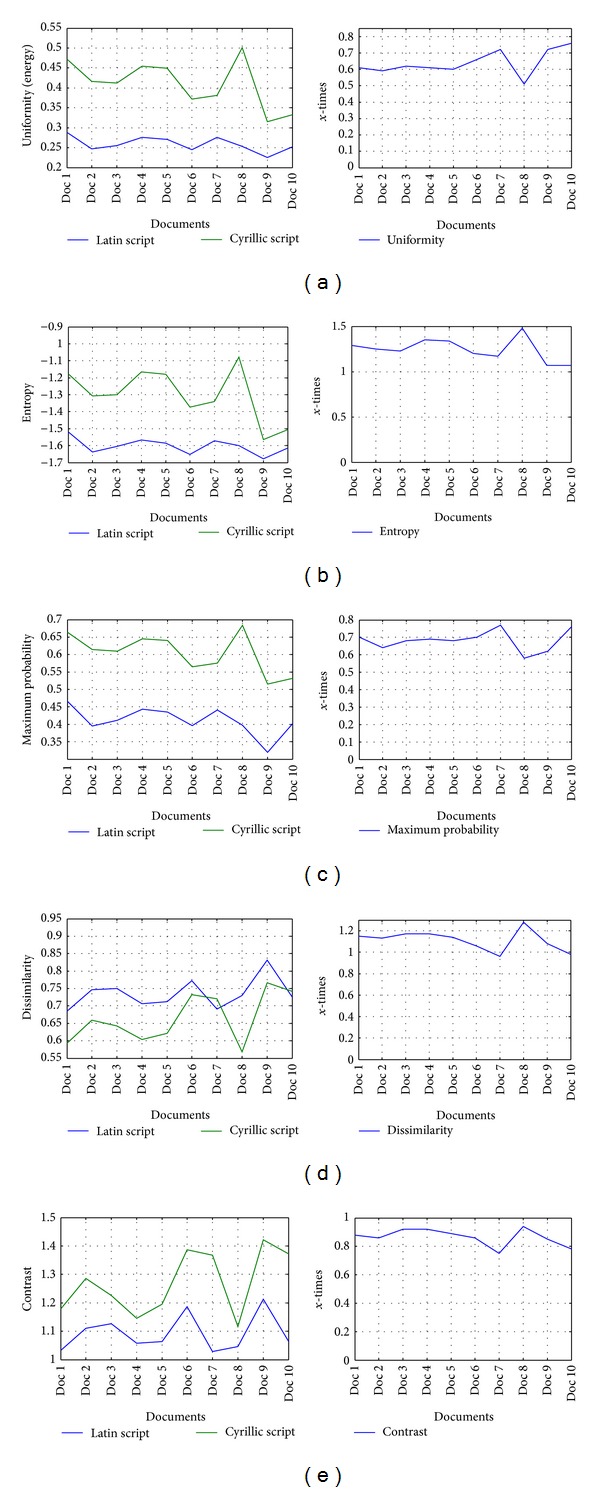
Illustrations of co-occurrence descriptors in Latin and Cyrillic text (left) and its ratio (right): (a) Uniformity, (b) Entropy, (c) Maximum probability, (d) Dissimilarity, (e) Contrast.

The co-occurrence descriptor for Latin and Cyrillic text and its ratio is presented in Figure 13.

From the above results, some criteria can be established. It is clear that uniformity and maximum probability receive the most distinct values in Latin and Cyrillic text. Hence, these descriptors are suitable for qualitative characterization of Latin and Cyrillic text as well as for creating criteria to distinguish a certain script type. From the above results, the margin criteria should be uniformity of 0.3 and maximum probability of 0.5. These values of both descriptors represent the strong margin in qualifying the script in certain Serbian text. If we accompany them with the criteria obtained from frequency analysis of the script type occurrence, then the full criteria of decision making can be established. This will lead to correct recognition of the script in Serbian text.

## 5. Conclusion

The paper proposed the algorithm for recognition of exact script in Serbian document. Documents in Serbian language can be written in two different scripts: Latin or Cyrillic. The proposed algorithm converts document written in Latin and Cyrillic script into cipher text. This way, all alphabetic characters are exchanged with only four different encrypted signs according to predefined encryption based on text line structure definition. Such ciphers texts are then subjected to the frequency and co-occurrence analysis. According to the obtained results a criteria for recognition of the certain script is proposed. The proposed method is applied to the custom-oriented database which includes different types of printed and web documents. The experiment shows encouraging results. Possible applications can be seen in the area of web page recognition. 

Future work will be toward the recognition of related languages as well as different languages written in the same script.

## Figures and Tables

**Figure 12:**
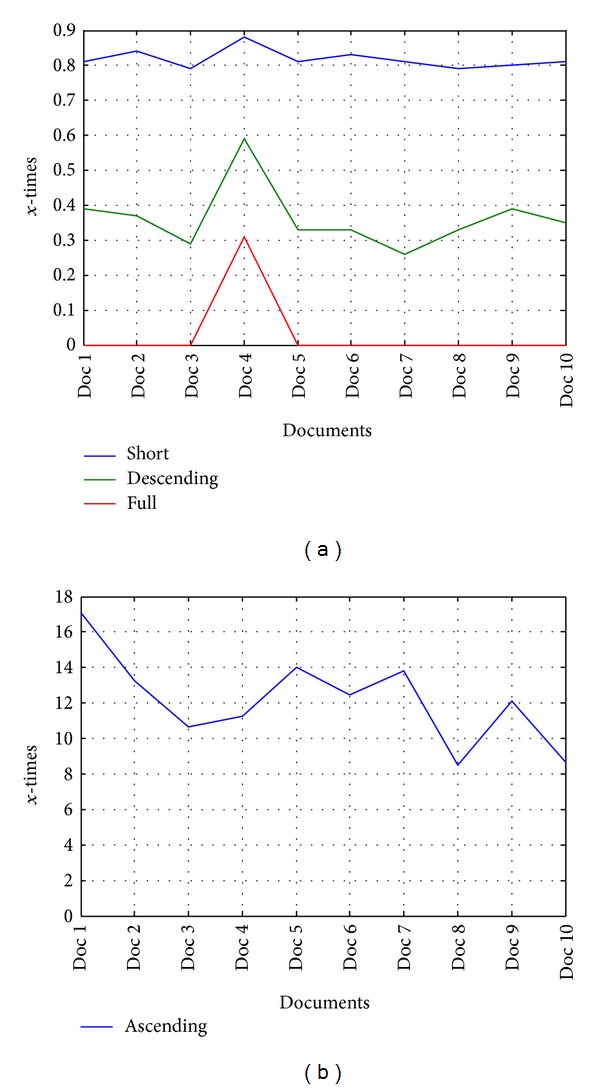
The ratio of the script type occurrence: (a) short, descending and full, (b) ascending.

**Table 1:** Definition of script types according to the baseline characteristics.

Script example	Type of script	Designation
a	Short	S
b	Ascender	A
j	Descender	D
lj	Full	F

**Table 2:** Serbian Latin and equivalent Cyrillic alphabet according to the script types.

Alphabet	Latin	Script types	Latin	Script types	Cyrillic	Script types	Cyrillic	Script types
1	A	A	a	S	*А*	A	*а*	S
2	B	A	b	A	*Б*	A	*б*	A
3	C	A	c	S	*Ц*	F	*ц*	D
4	Ć	A	ć	A	*Ћ*	A	*ћ*	A
5	Č	A	č	A	*Ч*	A	*ч*	S
6	D	A	d	A	*Д*	F	*д*	S
7	Đ	A	đ	A	*Ђ*	A	*ђ*	F
8	Dž	A	dž	A	*Џ*	F	*џ*	D
9	E	A	e	S	*Е*	A	*е*	S
10	F	A	f	A	*Ф*	A	*ф*	F
11	G	A	g	D	*Г*	A	*г*	S
12	H	A	h	A	*Х*	A	*х*	S
13	I	A	i	S	*И*	A	*и*	S
14	J	A	j	D	*Ј*	A	*ј*	D
15	K	A	k	A	*К*	A	*к*	S
16	L	A	l	A	*Л*	A	*л*	S
17	Lj	F	lj	F	*Љ*	A	*љ*	S
18	M	A	m	S	*М*	A	*м*	S
19	N	A	n	S	*Н*	A	*н*	S
20	Nj	F	nj	D	*Њ*	A	*њ*	S
21	O	A	o	S	*О*	A	*о*	S
22	P	A	p	D	*П*	A	*п*	S
23	R	A	r	S	P	A	*р*	D
24	S	A	s	S	*С*	A	*с*	S
25	Š	A	š	A	*Ш*	A	*ш*	S
26	T	A	t	A	*Т*	A	*т*	S
27	U	A	u	S	*У*	A	*у*	D
28	V	A	v	S	*В*	A	*в*	S
29	Z	A	z	S	*З*	A	*з*	S
30	Ž	A	ž	A	*Ж*	A	*ж*	S

**Table 3:** Statistical analysis of Latin and Cyrillic script types.

Script	Type of letters	Occurrence of script types				Distribution of script types			
		S	A	D	F	S (%)	A (%)	D (%)	F (%)
Latin	Capital letters	0	28	0	2	0	93.33	0	6.67
Latin	Small letters	12	13	4	1	40.00	43.33	13.33	3.34
Cyrillic	Capital letters	0	27	0	3	0	90.00	0	10.00
Cyrillic	Small letters	21	2	5	2	70.00	6.67	16.66	6.67

**Table 4:** Percentage of script type occurrence in document.

Type of script (TOS)	Latin	Cyrillic	*x* times
S	71.88%	82.76%	0.87
A	19.39%	1.74%	11.14
D	8.46%	14.64%	0.57
F	0.27%	0.86%	0.31

It is obvious that the Latin document compared to Cyrillic one has slightly smaller number of short (S), descender (D), and full (F) letters. Nonetheless, the crucial margin is seen in ascender (A) letters. Hence, it can be a measure of confidence for detection of the script in a document given in Serbian language.

**Table:** (a) For cipher text obtained from the Latin text

0.3722	0.2212	0.0623	0.0048
0.2220	0.0343	0.0072	0
0.0623	0.0072	0.0016	0
0.0048	0	0	0

**Table:** (b) For cipher text obtained from Cyrillic text

0.5863	0.0327	0.1326	0.0064
0.0391	0.0104	0.0144	0
0.1262	0.0200	0.0224	0.0016
0.0072	0	0.0008	0

**Table 6:** Cooccurrence descriptors for Latin and Cyrillic cipher text.

Serbian language	Latin	Cyrillic	Characterization
Uniformity (energy)	0.2459	0.3811	Latin < Cyrillic
Entropy	−1.6298	−1.4363	Latin > Cyrillic
Maximum probability	0.3722	0.5863	Latin < Cyrillic
Dissimilarity	0.7356	0.6669	Latin > Cyrillic
Contrast	1.0423	1.2660	Latin < Cyrillic

From the above results, it is clear that co-occurrence descriptors can fully characterize the difference between Latin and Cyrillic script. This means that frequency analysis of the occurrence can be supplemented with additional attributes in order to define a strong margin as a criterion to distinguish a certain script.

**Table 7:** Frequency analysis of the script type occurrence in documents from database.

Printed documents										
Type of script	Doc 1		Doc 2		Doc 3		Doc 4		Doc 5	
	Latin	Cyrillic	Latin	Cyrillic	Latin	Cyrillic	Latin	Cyrillic	Latin	Cyrillic
S	2243	2764	11396	13593	1510	1914	2217	2516	2069	2542
A	906	53	4060	306	693	65	598	53	897	64
D	183	468	724	1933	82	286	261	445	151	461
F	0	7	0	8	0	8	8	26	0	12

Web documents										
Type of script	Doc 6		Doc 7		Doc 8		Doc 9		Doc 10	
	Latin	Cyrillic	Latin	Cyrillic	Latin	Cyrillic	Latin	Cyrillic	Latin	Cyrillic

S	1486	1799	1358	1682	783	996	1657	2078	1328	1637
A	598	48	636	46	408	48	750	62	588	68
D	99	304	75	292	58	174	134	344	99	284
F	0	7	0	9	0	13	0	18	0	9

The above results are further processed in order to calculate the ratio of script type occurrence between Latin and Cyrillic document. Complete results are given in Table 8.

**Table 8:** The ratio of script type occurrence between Latin and Cyrillic documents.

Type of script	Doc 1	Doc 2	Doc 3	Doc 4	Doc 5	Doc 6	Doc 7	Doc 8	Doc 9	Doc 10
S	0.81	0.84	0.79	0.88	0.81	0.83	0.81	0.79	0.80	0.81
A	17.09	13.27	10.66	11.28	14.02	12.46	13.83	8.50	12.10	8.65
D	0.39	0.37	0.29	0.59	0.33	0.33	0.26	0.33	0.39	0.35
F	0.00	0.00	0.00	0.31	0.00	0.00	0.00	0.00	0.00	0.00

The results are presented in Figure 12.

**Table 9:** The ratio of script type occurrence measures.

Type of script	∑		Ratio			Criteria
	Latin	Cyrillic	Average	Max.	Min.	
S	26047	31521	0.82	0.88	0.79	>0.75
A	10134	813	12.21	17.09	8.50	>8
D	1866	4991	0.36	0.59	0.26	<0.6
F	8	117	0.03	0.31	0.00	?

**Table 10:** GLCM five descriptors of the script type co-occurrence in documents from database.

Printed documents										
	Doc 1		Doc 2		Doc 3		Doc 4		Doc 5	
	Latin	Cyrillic	Latin	Cyrillic	Latin	Cyrillic	Latin	Cyrillic	Latin	Cyrillic
Uniformity	0.2885	0.4725	0.2473	0.4167	0.2557	0.4120	0.2759	0.4545	0.2707	0.4498
Entropy	−1.5191	−1.1774	−1.6379	−1.3079	−1.6047	−1.2999	−1.5675	−1.1650	−1.5847	−1.1799
Max. probability	0.4655	0.6636	0.3952	0.6139	0.4120	0.6098	0.4439	0.6457	0.4349	0.6405
Dissimilarity	0.6847	0.5933	0.7469	0.6592	0.7502	0.6427	0.7064	0.6041	0.7117	0.6217
Contrast	1.0324	1.1790	1.1106	1.2859	1.1258	1.2261	1.0577	1.1449	1.0630	1.1949

Web documents										
	Doc 6		Doc 7		Doc 8		Doc 9		Doc 10	
	Latin	Cyrillic	Latin	Cyrillic	Latin	Cyrillic	Latin	Cyrillic	Latin	Cyrillic

Uniformity	0.2447	0.3714	0.2754	0.3817	0.2533	0.5005	0.2252	0.3147	0.2522	0.3325
Entropy	−1.6524	−1.3738	−1.5725	−1.3412	−1.5990	−1.0779	−1.6778	−1.5650	−1.6144	−1.5059
Max. probability	0.3964	0.5650	0.4409	0.5753	0.3972	0.6844	0.3195	0.5154	0.4016	0.5318
Dissimilarity	0.7723	0.7320	0.6912	0.7209	0.7294	0.5686	0.8317	0.7667	0.7256	0.7416
Contrast	1.1862	1.3869	1.0287	1.3681	1.0459	1.1158	1.2122	1.4220	1.0641	1.3716

The above results are further processed in order to calculate the ratio of script type co-occurrence in between Latin and Cyrillic document. These results are shown in Table 11.

**Table 11:** The ratio of the co-occurrence descriptors between Latin and Cyrillic documents.

	Doc 1	Doc 2	Doc 3	Doc 4	Doc 5	Doc 6	Doc 7	Doc 8	Doc 9	Doc 10
Uniformity	0.61	0.59	0.62	0.61	0.60	0.66	0.72	0.51	0.72	0.76
Entropy	1.29	1.25	1.23	1.35	1.34	1.20	1.17	1.48	1.07	1.07
Max. probability	0.70	0.64	0.68	0.69	0.68	0.70	0.77	0.58	0.62	0.76
Dissimilarity	1.15	1.13	1.17	1.17	1.14	1.06	0.96	1.28	1.08	0.98
Contrast	0.88	0.86	0.92	0.92	0.89	0.86	0.75	0.94	0.85	0.78

The final processing of the above results is based on cumulative measures like average, max. and min. of script type co-occurrence in the database. According to that certain criteria are established. All these are shown in Table 12.

**Table 12:** The ratio of script type co-occurrence descriptors.

	Latin			Cyrillic			Ratio			Criteria
	Min.	Max.	Average	Min.	Max.	Average	Max.	Min.	Average	
Uniformity	0.23	0.29	0.26	0.31	0.50	0.41	0.76	0.51	0.64	0.3
Entropy	−1.68	−1.52	−1.60	−1.57	−1.08	−1.30	1.48	1.07	1.25	?
Max. probability	0.32	0.47	0.42	0.52	0.68	0.60	0.77	0.58	0.68	0.5
Dissimilarity	0.68	0.83	0.74	0.57	0.77	0.67	1.28	0.96	1.11	?
Contrast	1.03	1.21	1.12	1.12	1.42	1.27	0.94	0.75	0.86	?
